# Perceived health-related quality of life in people living with HIV co-infected with SARS-CoV-2 in France

**DOI:** 10.1007/s11136-024-03701-4

**Published:** 2024-06-12

**Authors:** Yvenie Amboise, Issifou Yaya, Lisa. Yombo-Kokule, Guillaume Roucoux, Arnaud Nze Ossima, Marie Preau, James W. Griffith, Fabienne Marcellin, Olivier Chassany, Antoine Cheret, Martin Duracinsky

**Affiliations:** 1 Patient-Reported Outcomes Research (PROQOL), Health Economics Clinical Trial Unit (URC- ECO), Hotel-Dieu Hospital, AP-HP, Paris, France; 2 ECEVE, UMR-S 1123, Paris Cité University, Inserm, Paris, France; 3 Lyon 2 Lumière University, Inserm Unit 1296 Radiations : Defense, Health, Environment, Lyon, France; 4 Feinberg School of Medicine, Department of Medical Social Sciences, Northwestern University, Chicago Illinois, USA; 5 Aix Marseille Univ, Inserm, IRD, SESSTIM, Sciences Economiques & Sociales de la Santé & Traitement de l’Information Médicale, ISSPAM, Marseille, France; 6 Plateforme de Diagnostic et de Thérapeutique Pluridisciplinaire, CHU Guadeloupe, Les Abymes, France; 7 INSERM, U1016, CNRS, UMR8104, Institut Cochin, Paris, France; 8 Département de Médecine Interne et d’Immunologie Clinique, Hôpital Bicêtre, AP-HP, Le Kremlin-Bicêtre, France

**Keywords:** SARS-CoV-2, COVID-19, Health-related quality of life, People living with HIV/AIDS, France

## Abstract

**Purpose:**

We aimed to assess health-related quality of life (HRQL) and its correlates among people living with HIV/AIDS (PLWHA) co-infected with SARS-CoV-2 in France.

**Methods:**

This cross-sectional was study conducted among PLWHA co-infected with SARS-CoV-2. HRQL was measured using the four dimensions of the PROQOL-HIV scale. Factors associated with each dimension were identified using linear regression.

**Results:**

mean (SD) scores for HRQL dimensions: 76.7 (± 21.1) for Physical Health and Symptoms (PHS), 79.2 (± 23.6) for Social Relationships (SR), 67.3 (± 27.4) for Mental and Cognitive (MC), and 83.9 (± 16.5) for Treatment Impact (TI). Employment status and COVID-19 knowledge were associated with higher PHS score, while blood transfusion-acquired HIV, CDC HIV, hospital discharge instructions, and self-reported symptoms were associated with lower PHS score. Couple status was associated with higher SR score, whereas, hospital discharge instructions, CDC HIV stage C, drug injection-acquired HIV, self-reported symptoms, and COVID-19 vulnerability perception were associated with lower SR score. Employment status and French birth were associated with higher MC score, while female sex, detectable HIV viral load, hospital discharge instructions, COVID-19 vulnerability perception, smoking, and self-reported symptoms were associated with lower MC score. French birth and homosexual/bisexual relationships-acquired HIV were associated with higher TI score, while detectable HIV viral load, psychiatric disorders, and self-reported symptoms were associated with lower TI score

**Conclusion:**

Among PLWHA co-infected with SARS-CoV-2, the scores of HRQL were impaired, particularly in the MC dimension. Findings underscore the multidimensional nature of HRQL, with notable variations across different dimensions. Understanding these correlates is crucial for tailored interventions aimed at improving the well-being of this population.

## Introduction

COVID-19, an infectious disease due to a Severe Acute Respiratory Syndrome coronavirus (SARS-CoV-2) [1] caused several millions of deaths in the world. Since March 2020, COVID-19 has been considered as a pandemic [2], and has negatively and dramatically impacted all areas of socioeconomic life. The COVID-19 pandemic affected all regions of the world disproportionately, with higher numbers of cases and deaths in developed countries [3]. Despite the progressive development of effective prevention strategies and measures, such as masking, physical distancing, or vaccination [3], the COVID-19 pandemic significantly impacted certain vulnerable groups, especially people living with comorbidities that maintain a chronic systemic inflammatory state [4, 5]. In the general population, cardiovascular diseases (including hypertension and diabetes), chronic kidney diseases, and cancers were the main comorbidities reported to be associated with both COVID-19 severity and a higher risk of death [6]. People living with HIV/AIDS (PLWHA), due to their immune status, are the most likely to have serious consequences related to the presence of comorbidities [4, 7]. In the early phases of the COVID-19 pandemic, several studies, including systematic reviews on the prevalence, have been conducted on clinical and psychosocial issues related to COVID-19 and HIV infection [8–10]. However, the impact of COVID-19 on PLWHA has not been fully evaluated yet [11]. In high-income countries, even if the good organization of the health care system has made it possible to avoid a total rupture of health care services related to HIV infection [12], studies in PLWHA reported an important psychological impact [13–15] .

Health-related quality of life (HRQL) may be affected in the context of the COVID-19 pandemic due to a lack of social connection, financial stress, or existing physical or mental health issues [16, 17]. In previous studies, findings demonstrated the multidimensional impact of the COVID-19 pandemic on different population groups, with most of the negative impacts being borne by vulnerable people, including PLWHA [18, 19]. Studies, conducted in various settings, highlighted that COVID-19 negatively affects all dimensions of HRQOL among adults patients regardless HIV status [19–23].

Measuring HRQL is needed to evaluate the impact of HIV and SARS-CoV-2 coinfection on general, physical and mental perceived health. HRQL assessment may help caregivers identify modifiable factors and integrate them into care strategies to improve the HRQL of COVID-19 survivors among PLWHA.

Recent studies, which COVID-19 patients involved in a pulmonary rehabilitation program (PRP) in Chile [24], and parents of adolescents in Norway [25] reported worse HRQL scores during the COVID-19 pandemic. Using WHOQOL-BREF scale, a study among PLWHA in India during COVID era found an average overall score for the HRQL of 51.35, including in physical health (54.39), psychological, (44.85), social relationships (48.48) and environment (57.69) domains [26]. In addition, during the first lockdown, the COVIPACT study conducted among French patients receiving treatment for solid or hematological cancers, found that 21% experienced symptoms of PTSD that were found to be associated with lower HRQL scores [27]. In a prospective observational study in France, which included, in a PRP, COVID-19 survivor patients found low mean scores in mental dimensions (40 ± 10) and physical dimensions (33 ± 11) of HRQL at the baseline [28]. Through scientific literature, socio-demographic characteristics, clinical features, or behavioral factors may influence HRQL among PLWHA during the COVID-19 pandemic.

To our knowledge, no published studies in France have assessed the HRQL of PLWHA co-infected with SARS-CoV-2. Thus, this explorative study aimed to document HRQL in PLWHA co-infected with SARS-CoV-2 and to determine the factors associated with its different dimensions.

## Methods

### Design of the COVIDHIV cohort

COVIDHIV is a French, multicenter, observational cohort study aiming at describing the clinical and biological features of the COVID-19 disease in PLWHA. Documenting patient-reported outcomes (including HRQL) among PLWHA with COVID-19 disease was one of the secondary objectives of the cohort study. From March 2020 to June 2022, adult PLWHA with confirmed infection with SARS-CoV-2 since January 1st, 2020, with and without criteria of hospitalization, were recruited in 42 hospitals located throughout the national territory, then followed up for one year. Infection with SARS-CoV-2 was confirmed using Polymerase Chain Reaction (PCR) test, serological test, imaging (CT scan or radiography), or antigenic test. PLWHA known to be co-infected with SARS-CoV-2 since January 2020 were included retrospectively, and those with a positive SARS-CoV-2 diagnostic test were included prospectively within seven days of the positive test.

Non-inclusion criteria were as follows: not speaking French, being under 18 years of age, being pregnant or breastfeeding, having a confirmed diagnosis of a pathogen other than SARS-CoV-2, being under guardianship or trusteeship mandate for future protection, participating in another study without the sponsor’s consent, not being a beneficiary or entitled to a social security scheme or state medical aid.

### Data collection

Clinical, biological and therapeutic data were collected from patient records using an electronic case report from (eCRF). Socio-behavioral data and patient-reported outcomes, including HRQL, were collected using self-administered questionnaires.

The questionnaires also included a specific module developed to assess knowledge, beliefs, and behaviors in the context of COVID-19. The corresponding items were tested on five patients during a group debriefing prior to implementation.

### Study population

In the present study, we analyzed the data collected at inclusion for all cohort participants (study population).

### Outcome measurement(s)

The variable of interest in this study is HRQL, measured using the PROQOL-HIV scale [29] reduced to the following four dimensions [30]: Physical Health and Symptoms (PHS: 11 items); Social Relationships (SR: 7 items); Mental and Cognitive (MC: 10 items); Treatment Impact (TI: 10 items). For each dimension, a score ranging from 0 (poorest HRQL) to 100 (best HRQL) was calculated. The PROQOL-HIV scale was validated in 11 local languages, including French [29, 30].

On the basis of the scientific literature, we tested potential explanatory variables among sociodemographic, behavioral, clinical, and COVID-19-related characteristics of individuals. Tested sociodemographic characteristics included age, sex, educational level, occupation, country of birth (born in metropolitan France (no/yes)), and living in a couple. Behavioral characteristics focused on the use of psychoactive substances: tobacco or e-cigarettes, alcohol (calculated from the AUDIT-C score: greater or equal to 3 for women and greater or equal to 4 for men determined unhealthy alcohol use [31]). HIV-related characteristics included time since diagnosis, transmission mode, CDC clinical stage, CD4 T-cell count (in cells/mm^3^), plasma viral load detectability (> 50 copies/mL), and the number of self-reported symptoms (experienced during the past two weeks), assessed using the 23-item modified HIV Symptom Index [32]. Tested clinical characteristics also included comorbidities, including the presence of overweight or obesity (body mass index (BMI) ≥ 25), psychiatric disorders, respiratory diseases, cardiovascular diseases, hypertension, diabetes, and cerebrovascular diseases, as well as hepatitis B virus (HBV) or hepatitis C virus (HCV) co-infection. Tested COVID-19-related characteristics included hospitalization for COVID-19, COVID-19 reinfection, time between confirmed COVID-19 diagnosis and inclusion, receipt of psychological support (“*Have you received any psychological support in the last two weeks?*”), individuals’ receipt of clear instructions at hospital discharge, as well as their perceptions regarding their level of knowledge about COVID-19 and their vulnerability to this disease. The latter three characteristics were assessed using the following items: *“When you were discharged from the hospital, did you receive clear instructions about precautions to be taken and further treatment?”, “Do you think you have sufficient knowledge about COVID-19?”, “Do you feel more vulnerable to COVID-19 because of your HIV status?”.* For each of these items, individuals had to indicate their level of agreement on a 5-answer Likert scale. We considered the answers *“I agree”* and *“I totally agree”* as denoting agreement with the item wording (vs. *“I do not agree at all”, “I do not agree” and “unconcerned”*).

In addition, we adjusted our analyses for the COVID-19 wave period in France (from March 2020 to September 2020 for the 1st, from September 2020 to March 2021 for the 2nd, from March 2021 to July 2021 for the 3rd, from July 2021 to October 2021 for the 4th and from October 2021 to July 2022 for the 5th wave).

### Statistical analyses

We performed a descriptive analysis of the study population’s characteristics and HRQL scores at inclusion in the COVIDHIV cohort. Continuous variables were expressed as mean (± standard deviation (SD)) and/or median (interquartile range (IQR)), and categorical variables as number (percentage).

Then, univariable analyses were performed using linear regression models to identify factors associated with the four HRQL scores (i.e. PHS, MC, SR, and TI). For each score, variables with a p-value ≤ 0.25 in the univariable analyses were included in the multivariable analysis.

A multivariable linear regression model was built for each HRQL score, in which the crude and adjusted β coefficients and associated 95% confidence intervals (CI) were estimated for all explanatory variables. Backward selection was used to identify potential associated factors, and variables with a p-value less than or equal to the significance threshold of 5% were retained in the final model for each score. The assumptions of linearity, normality, homogeneity, and independence of the residuals were checked before the synthesis of the results. Finally, to maintain good power in our analyses, missing data were categorized as “not reported” and coefficients were not presented in the table. The R Studio/4.1.3 software was used for all the analyses [33].

## Results

### Main characteristics of the study population

A total of 371 individuals (24.1% prospective cases and 75.9% retrospective cases of SARS-CoV-2 infection) were included, among who 64.7% were male. The median (IQR) age was 53 (44.0–60.0) years, 62.5% of the participants were professionally active, 44.1% were born in metropolitan France, 52.8% were living in couple, 85.1% had secondary education or more. In addition, 35.1% of the participants reported unhealthy alcohol use, and 17.7% reported smoking tobacco or e-cigarettes (Table 1).

**Table 1:** Sociodemographic, clinical, biological and behavioral characteristics of PLWHA co-infected with SARS-CoV-2 followed-up in French hospitals: data at inclusion in the COVIDHIV cohort (*n* = 371) J’ai surligné en rouge les variables ayant un fort % de valeurs manquantes, qui ne sont pas exploitables en l’état (hormis peut-être la variable réinfection, car elle ne rentre pas dans les multivariées)

Characteristics (% of missing values)	Median (IQR) or *n* (%)	
**Sociodemographic characteristics**		
Age (years) (0)	53 (44.0–60.0)	
Sex, *female* (0)	131 (35.3)	
High school level or higher (11.6)	279 (85.1)	
Professionally active (2.2)	227 (62.5)	
Born in metropolitan France (< 0.1)	163 (44.1)	
Living in a couple (2.9)	190 (52.8)	
**HIV-related characteristics**		
Heterosexual transmission (2.4)	185 (51.1)	
Homosexual or bisexual transmission (2.4)	142 (39.2)	
Transfusion transmission (2.4)	5 (1.4)	
Drug injection transmission (2.4)	19 (5.2)	
CD4 count ≥ 500 cells/mm^3^ (1.6)	236 (64.7)	
Detectable HIV viral load (> 50 copies/mL) (1.9)	30 (8.2)	
CDC HIV clinical stage* (1.6) *A* *B* *C*	194 (54.0) 62 (17.3) 103 (28.7)	
Time since HIV diagnosis (years) (12.7)	17.0 (8.0–24.0)	
Number of self-reported symptoms (14.0)	7.0 (3.0–11.0)	
**COVID-19-related characteristics**		
Hospitalization for COVID-19 (4.3)	49 (13.8)	
COVID-19 reinfection (41.2)	16 (7.3)	
Time between confirmed COVID-19 diagnosis and inclusion (days) (0.5)	35.0 (9.0–77.0)	
Receipt of psychological support (15.4)	108 (34.4)	
Receipt of clear instructions at hospital discharge (32.3)	184 (73.3)	
Perceived knowledge about COVID-19 (14.8)	183 (57.9)	
Perceived vulnerability to COVID-19 (15.0)	168 (53.3)	
COVID-19 wave** (0) *1st* *2nd* *3rd* *4th* *5th*	149 (40.2) 107 (28.8) 40 (10.8) 10 (2.7) 65 (17.5)	
**Comorbidities**		
HBV coinfection (3.0)	18 (5.0)	
HCV coinfection (3.2)	10 (2.8)	
Psychiatric disorders (5.4)	53 (15.1)	
Respiratory diseases (5.1)	25 (7.1)	
Cardiovascular diseases (5.1)	121 (34.4)	
Hypertension (67.6)	100 (83.3)	
Diabetes (5.1)	43 (12.2)	
Overweight or obesity*** (9.4)	190 (56.5)	
Unhealthy alcohol use (43.9)	73 (35.1)	
Tobacco or e-cigarette smoking (14.6)	56 (17.7)	

Concerning HIV-related characteristics, the median (IQR) time since HIV diagnosis was 17.0 (8.0–24.0) years, 28.7% of individuals had a CDC clinical stage C; 64.7% had a CD4 count ≥ 500 cells/mm^3^; and 8.2% had a detectable HIV viral load. The median number of self-reported symptoms was 7.0 (3.0–11.0).

Regarding COVID-19-related characteristics, 49 participants (13.8%) had been hospitalized. Only 7.3% of the participants were reinfected by SARS-CoV-2. In addition, 34.4% had received psychological support, 73.3% received clear instructions at hospital discharge (data available for 67.6% of the participants), 53.3% perceived themselves as more vulnerable to COVID-19 because of HIV, and 57.9% considered they had sufficient knowledge about COVID-19 (Table 1).

### HRQL of participants

The distributions of scores associated with the four PROQOL-HIV dimensions are presented in Table 2. The highest mean and median scores were found in the TI dimension, while the lowest were found in the MC dimension.

**Table 2:** Health-related quality of life among people living with HIV co-infected with SARS-CoV-2 in France: data at inclusion in the COVIDHIV cohort study (*n* = 371)

HRQL scores*	*N*	Median (IQR)	Mean (± SD)
Physical health and symptoms (PHS)	312	81.8 (61.4, 95.5)	76.4 (± 21.1)
Social relationships (SR)	314	85.7 (64.3, 100.0)	78.3 (± 23.9)
Mental and Cognitive (MC)	313	72.5 (47.5, 90.0)	66.5 (± 26.9)
Treatment impact (TI)	309	90.0 (77.5, 95.0)	82.7 (± 17.1)

HRQL: health-related quality of life; IQR: interquartile range; SD: standard deviation

* PROQOL-HIV scale (20)

### Factors associated with HRQL scores

Factors associated with each of the four dimensions of PROQOL-HIV are presented in Table 3.

**Table 3:** Factors associated with HRQL dimensions (PROQOL-HIV scores) among PLWHA co-infected with SARS-CoV-2 in France (linear regression models, COVIDHIV cohort, *n* = 371)

Characteristics	Physical Health and Symptom (PHS)		Social Relationships (SR)		Mental and Cognitive (MC)		Treatment Impact (TI)	
	Univariable Coef [95% CI]	Multivariable^Ψ^ Coef [95% CI]	Univariable Coef [95% CI]	Multivariable^Ψ^ Coef [95% CI]	Univariable Coef [95% CI]	Multivariable^Ψ^ Coef [95% CI]	Univariable Coef [95% CI]	Multivariable^Ψ^ Coef [95% CI]
**Sociodemographic characteristics**								
Sex, female	-7.92 [-12.78 ; -3.06]		-6.40 [-11.93 ;-0.87]		-17.25 [-23.24 ; -11.26]	-8.04 [-13.48 ; -2.59]	-6.22 [-10.19 ; -2.25]	
High school level or higher	8.34 [1.17 ; 15.52]		10.24 [2.22; 18.27]		10.49 [1.44 ; 19.54]		-0.08 [-5.93 ; 5.77]	
Professionally active	7.10 [2.29 ; 11.91]	4.77 [0.98 ; 8.55]	8.09 [2.70 ; 13.48]		8.30 [2.15 ; 14.46]	5.81 [0.64 ; 10.97]	2.13 [-1.87 ; 6.12]	
Born in metropolitan France	9.48 [4.88 ; 14.08]		6.41 [1.14 ; 11.69]		18.83 [13.20 ; 24.46]	12.10 [6.84 ; 17.35]	6.84 [3.06 ; 10.61]	4.42 [-0.26 ; 8.58]
Living in a couple	2.09 [-2.70 ; 6.87]		6.82 [1.48 ; 12.15]	5.67 [0.90 ; 10.44]	7.05 [1.01 ; 13.08]		0.06 [-3.86 ; 3.97]	
**Psychoactive substances use**								
Unhealthy alcohol use	-1.05 [-7.12 ; 5.02]		-0.06 [-6.91 ; 6.79]		2.66 [-4.79 ; 10.11]		2.45 [-2.47 ; 7.37]	
Tobacco or e-cigarette smoking	-1.09 [-7.33 ; 5.15]		-5.98 [-13.05 ; 1.08]		-5.23 [-13.10 ; 2.64]	-10.57 [-17.09 ; -4.05]	-3.36 [-8.39 ; 1.67]	
**HIV-related characteristics**								
Heterosexual transmission	-4.04 [-8.77 ; 0.69]		-3.36 [-8.70 ; 1.98]		-15.33 [-21.13 ; -9.52]		-6.80 [-10.60 ; -3.00]	
Homosexual or bisexual transmission	7.05 [2.28 ; 11.83]		7.61 [2.22 ; 13.00]		17.97 [12.13 ; 23.80]		8.97 [5.16 ; 12.79]	6.70 [2.68 ; 10.71]
Transfusion transmission	-14.77 [-33.48 ; 3.95]	-15.31 [-29.22 ; -1.39]	-11.21 [-32.39 ; 9.98]		-16.71 [-40.59 ; 7.16]		-4.23 [-19.47 ; 11.01]	
Drug injection transmission	-10.57 [-20.62 ; -0.52]		-17.67 [-29.25 ; -6.09]	-14.29 [-24.94 ; -3.65]	-5.36 [-18.25 ; 7.53]		-3.98 [-12.18 ; 4.23]	
CD4 count ≥ 500 cells/mm^3^	-0.40 [-5.40 ; 4.61]		3.15 [-2.47 ; 8.77]		4.55 [-1.78 ; 10.87]		3.12 [-0.96 ; 7.20]	
Detectable HIV viral load (> 50 cp/mL)	0.64 [-8.21 ; 9.49]		-4.31 [-14.30 ; 5.69]		-12.77 [-23.97 ; -1.57]	-11.50 [-21.16 ; -1.84]	-10.60 [-17.83 ; -3.36]	-11.43 [-18.74 ; -4.11]
CDC HIV clinical stage (ref = A) B C	-6.92 [-13.32 ; -0.53] -9.83 [-15.35 ; -4.30]	-5.98 [-10.94 ; -1.02] -7.48 [-11.78 ; -3.17]	-9.81 [-17.04 ; -2.58] -9.21 [-15.43 ; -2.99]	-5.70 [-12.29; 0.89] -7.13 [-12.78 ; -1.48]	-8.22 [-16.45 ; 0.01] -5.39 [-12.48 ; 1.69]		-3.59 [-8.95 ; 1.77] -1.42 [-5.99 ; 3.15]	
Number of self-reported symptoms	-2.69 [-3.05 ; -2.33]	-2.52[-2.88 ; -2.16]	-2.00 [-2.48 ; -1.53]	-1.72 [-2.21 ; -1.24]	-1.93 [-2.48 ; -1.37]	-1.44 [-1.94 ; -0.93]	-0.82 [-1.19 ; -0.45]	-0.83 [-1.20 ; -0.46]
**COVID-19-related characteristics**								
Hospitalization for COVID-19	-4.73 [-12.99 ; 3.52]		-9.62 [-18.75 ; -0.48]		-7.64 [-17.66 ; 2.38]		-5.20 [-11.58 ; 1.17]	
New COVID-19 reinfection	6.91 [-5.03 ; 18.85]		-0.07 [-13.58 ; 13.44]		-6.64 [-21.79 ; 8.51]		-8.96 [-18.50 ; 0.57]	
Psychological support	-8.22 [-13.12 ; -3.32]		-4.48 [-9.99 ; 1.04]		-9.88 [-16.12 ; -3.64]		-3.42 [-7.46 ; 0.62]	
Receipt of clear instructions at hospital discharge	-5.97 [-11.89 ; -0.05]	-5.90 [-10.39 ; -1.41]	-7.34 [-13.94 ; -0.74]	-6.52 [-12.36 ; -0.67]	-7.78 [-15.33 ; -0.23]	-6.60 [-12.67 ; -0.53]	-2.88 [-7.71 ; 1.96]	
Perceived knowledge about COVID-19	6.08 [1.33 ; 10.83]	4.18 [0.56 ; 7.81]	-0.39 [-5.80 ; 5.02]		4.40 [-1.68 ; 10.49]		1.30 [-2.61 ; 5.21]	
Perceived vulnerability to COVID-19	-11.13 [-15.73 ; -6.54]		-12.06 [-17.24 ; -6.87]	-7.68 [-12.52 ; -2.84]	-23.61 [-29.05 ; -18.17]	-14.97 [-20.21 ; -9.73]	-7.71 [-11.49 ; -3.93]	
**Comorbidities**								
Psychiatric disorder	-4.11 [-10.84 ; 2.61]		-14.18 [-21.52 ; -6.84]		-4.31 [-12.80 ; 4.18]		-5.63 [-11.02 ; -0.24]	-6.58 [-11.85 ; -1.32]
Respiratory diseases	-9.43 [-18.59 ; -0.27]		-14.12 [-24.39 ; -3.85]		-2.00 [-13.73 ; 9.73]		-4.27 [-11.75 ; 3.21]	
Cardiovascular diseases	-4.39 [-9.45 ; 0.66]		-2.88 [-8.60 ; 2.84]		-1.11 [-7.55 ; 5.33]		-1.52 [-5.65 ; 2.62]	
Diabetes	-4.35 [-11.83 ; 3.12]		-2.82 [-11.25 ; 5.61]		0.48 [-8.93 ; 9.89]		1.00 [-5.02 ; 7.02]	
Overweight or obesity	-1.18 [-6.13 ; 3.76]		1.23 [-4.34 ; 6.79]		-5.65 [-11.89 ; 0.59]		-2.41 [-6.42 ; 1.61]	

Ψ Adjusted for age ; time between confirmation COVID-19 and inclusion ; COVID-19 wave Coef: regression coefficient; CI: confidence interval ; HRQL: health-related quality of life ; PLWHA: people living with HIV/AIDS

#### Physical health and symptoms (PHS)

Participants who were professionally active (β = 4.77, 95% CI: 0.98; 8.55) and those who reported having sufficient knowledge about COVID-19 (β = 4.18, 95%CI: 0.56; 7.81) had reported better scores in the PHS dimension. In contrast, participants who had acquired HIV by blood transfusion (β= -15.31; 95% CI: -29.22; -1.39), those who were at CDC stage B (β= -5.98; 95% CI: -10.94; -1.02) or stage C (β= -7.48; 95% CI: -11.78; -3.17) of CDC HIV classification, those who reported a higher number of symptoms(β= -2.52; 95% CI: -2.88; -2.16), and those who received clear instructions at discharge (β= -5.90; 95% CI: -10.39; -1.41) had a worse score in the PHS dimension (Table 3).

#### Social relationships (SR)

Participants living in couple (β = 5.67; 95% CI: 0.90; 10.44) had better scores in the SR dimension. In contrast, participants who perceived themselves vulnerable to COVID-19 (β= -7.68; 95% CI: -12.52; -2.84), those who were at CDC stage C (β= -7.13; 95% CI: -12.78; -1.48), those who had acquired HIV through drug injection (β= -14.29; 95% CI: -24.94; -3.65), those who reported a higher number of symptoms (β= -1.72; 95% CI: -2.21; -1.24), and those who received clear instructions at hospital discharge (β= -6.52; 95% CI: -12.36; -0.67) had a worse score for the SR dimension (Table 3).

#### Mental and cognitive (MC)

Participants who were professionally active (β = 5.81; 95% CI: 0.64; 10.97), those who were born in metropolitan France (β = 12.10; 95% CI: 6.84; 17.35), had better scores in the MC dimension. In contrast, women (β= -8.04; 95% CI: -13.48; -2.59), participants who had detectable HIV viral load (β= -11.50; 95% CI: -21.16; -1.84), those who reported a higher number of symptoms (β= -1.44; 95% CI: -1.94; -0.93), those who received discharge instructions (β= -6.60; 95% CI: -12.67; -0.53), those who perceived themselves as more vulnerable to COVID-19 (β= -14.97; 95% CI: -20.21; -9.73), and those who smoked (β= -10.57; 95% CI: -17.09; -4.05) had lower scores in the MC dimension (Table 3).

#### Treatment impact (TI)

Participants born in metropolitan France (β = 4.42; 95% CI: -0.26; 8.58), and those who had acquired HIV in homosexual or bisexual relationships (β = 6.70; 95% CI: 2.68; 10.71) had better scores in the TI dimension. In contrast, those who had a detectable HIV viral load (β= -11.43; 95% CI: -18.78; -4.11), those who reported a higher number of symptoms (β= -0.83; 95% CI: -1.20; -0.46), and those with psychiatric disorders (β= -6.58; 95% CI: -11.85; -1.32) had worse scores in the TI dimension (Table 3).

## Discussion

This nationwide multicenter study investigated HRQL in PLWHA co-infected with SARS-CoV-2. This study showed poor scores on all dimensions of HRQL, including PHS, SR, MC, and TI scores. In the four dimensions of HRQL, the MC dimension had the lowest mean score and the TI dimension had the highest mean score. The score for the TI dimension could be explained by a better adaptation of the structural organization of healthcare that ensures continuity of care for PLHWA, including HIV-related service delivery and quality of healthcare for PLWHA in the context of the COVID-19 pandemic [26, 34]. However, given the difficulties of supply of HIV treatment and the fear of exposure to COVID-19 for some patients, health care delivery, referral, and follow-up of the PLWH are also affected [35].

During the COVID-19 pandemic, the strategies and measures implemented to slow down its spread, including social restrictions and lockdown, leaded to an increase in stress, anxiety, and depression. It also caused considerable daily illnesses, unemployment, and economic hardship, which would lead to psychological health problems and barriers to health. In several studies on HRQL conducted in France prior the COVID-19 pandemic, the mental dimension remained the most altered [36, 37]. Studies in many countries have shown the negative impact of the COVID-19 pandemic on mental health among people regardless of COVID-19 or HIV status [38–40]. It is important for healthcare providers to take a holistic approach to care, addressing the physical and mental health needs of their patients. This may include providing counseling and support for mental health concerns, as well as ensuring access to HIV prevention and treatment services. In addition, PLWHA with COVID-19 can benefit from peer support groups, which can provide a sense of community and connection during this difficult time. This study showed that the consequences of COVID-19 on the mental health of PLWHA have been considerable while this population faced many situations impacting their mental health, among which stigmatization and discrimination related to HIV status [41, 42].

The COVID-19 pandemic impacted not only the TI and MC dimensions but also the SR dimension, which may due to a fear of exposure to COVID-19, health restrictions, etc. The PHS dimension was also affected, as for symptomatic patients, social restrictions may lead to a diminution of physical activity.

Even if we observed overall relative low scores for the HRQL dimensions, it is important to mention that this could hide certain heterogeneities related mainly to the characteristics of the participants, including sociodemographic characteristics, psychoactive substances use, HIV and COVID-19-related characteristics, and comorbidities. In fact, these findings highlight the complex interplay between socio-demographic, clinical, and psychosocial factors in shaping different dimensions of perceived HRQL among individuals co-infected with HIV and SARS-CoV-2 in France. Understanding these associations can inform targeted interventions aimed at improving overall well-being and treatment outcomes in this population.

This study highlighted the negative impact of the number of self-reported symptoms on all the dimensions of HRQL. Even though there were not COVID-19-specific symptoms, it is well known that people with chronic inflammatory conditions such as PLWHA [5, 43, 44] are considered at high risk of severe outcomes of COVID-19 or persistent symptoms. Moreover, the number of symptoms would constitute a proxy for comorbidities and, by far, a severity of COVID-19 in these participants. However, as demonstrated by Lechien J.R et al., the COVID-19-related symptoms may vary significantly with the age and gender of the patients [45] and exacerbate their impact on HRQL or well-being. Besides, participants who have a high number of symptoms would certainly be those with comorbidities and would therefore be much more vulnerable to SARS-CoV-2 infection or the severe form of COVID-19, including because of their HIV status.

In the same line, clinically, it was observed in our study that PLWHA who had a detectable HIV viral load or those at CDC stage B or C had worse HRQL. This is consistent with recent studies that have shown that PLWHA with CD4 counts above 500 cells/mm3 or those with detectable viremia would have a higher risk of acquiring SARS-CoV2 than the general population [46, 47]. . This double burden of COVID-19 and HIV infection would have a significant impact on their HRQL, which may be due to the immunological disturbance. Despite considerable progress in the management of HIV infection and the dramatic increase in life expectancy of PLWHA, almost comparable to that of an HIV seronegative individual, there remains a vulnerability due to chronic inflammation [44] and poor adherence that could compromise immune balance. This vulnerability may not only be experienced but also self-perceived by some participants.

In our study, because of their sero-status, participants who perceived themselves vulnerable to SARS-CoV-2 infection had worse HRQL, particularly in the MC and SR dimensions. This group may have better knowledge about COVID-19, including its modes of transmission and prevention strategies, and might be anxious, depressed, and stressed, fearing being infected by SARS-CoV-2 in the absence of effective treatment and vaccines [48]. Hence the importance of support in the care of this population, such as awareness campaigns and online information accessible to all PLWHA, in order to reduce the stress of patients.

Our study also showed that another aspect of support might be beneficial for the participants, as those living in couple reported better HRQL, mainly in the SR dimension. According to the study of Li X. and al., social support has shown a strong potential to influence quality of life [49]. Although our study does not address all of the supports for this population, it does provide information on some of the key supports that could affect the well-being of PLWHA. In our study, PLWHA who reported they receiving clear instructions from hospital staff at discharge, reported a poor score on the PHS, SR, and MC dimensions. The hypothesis would be that discharge instructions might be given to the most vulnerable and those who would have requested them via questioning. In this context, health care providers are recognized as an important source of psychological support [8]. This might be crucial for the well-being of the PLWHA group [14], who need close attention for an early diagnosis of further emotional distress. Apart from the COVID-19 pandemic, PLWHA may face mental health issues because of the stigmatization and discrimination [42, 50] in spite of the measures taken [41]. It is necessary to avoid any chronic worsening of mental health, which can have important repercussions on PLWHA [15]. Developing and implementing tailored psychosocial support programs for individuals co-infected with HIV and SARS-CoV-2. These programs could address the unique challenges and stressors faced by this population, such as stigma, social isolation, and concerns about dual infection management.

Besides, our study showed that participants who were professionally active had better scores in the PHS and MC dimensions. Being professionally active may offer a great opportunity to be mobile, in particular outside the period of lockdown. Breaking with measures of social and physical distancing should allow for the improvement of mental and physical well-being, through contact, exchanges with peers, colleagues, and community.

Being born in metropolitan France was associated with better scores in the MC and TI dimensions. Even if in the general population, individuals from sub-Saharan Africa reported better mental HRQL [37], in many other context, such as the COVID-19 pandemic, ethnic minorities are recognized as vulnerable to COVID-19 [51, 52], mostly because of their low health literacy and their precarious life conditions [51, 53], including the high prevalence of comorbidities [54]. Participants born abroad may come from diverse socioeconomic backgrounds compared to those born in Metropolitan France. Variations in income, education level, employment opportunities, and access to healthcare services can influence HRQL scores. Moreover, cultural norms, values, and social support systems can affect perceptions of health and quality of life. Consistent with our study, Drewes and al. underlined that infection, social circumstances, relationship problems, comorbidities (including hypertension), and stigma could have negatively impacted the HRQL of PLWHA [55].

This study has two main limitations. First, this is an observational study carried out using self-reported questionnaires; there could thus be a social desirability bias. However, participants were invited to answer with sincerity, and they were informed that their responses would be analyzed in a non-judgmental way. Second, this was a multicenter study, but we did not test for a possible center effect, given the very small number of participants in some centers (most of which had only one individual included). Given the convenience sampling, this result cannot be extrapolated to all the PLWHA in France or elsewhere.

## Conclusion

We found in this study that the HRQL in PLWHA co-infected with SARS-CoV-2 in France was impaired, particularly in the Mental and Cognitive dimension. Several factors, including clinical features (the number of self-reported symptoms, the clinical stage of HIV infection, HIV viral load), professional activity, living in a couple, perceived vulnerability to COVID-19, being born in France, and receiving clear instructions at hospital discharge, are associated with the HRQL in this population at the time of enrolment. Our results offer an opportunity to better inform and educate PLWHA about COVID-19 and caregivers in the context of the pandemic through a combination of stress management and empowerment interventions at the individual, local, national, and international levels. Conducting longitudinal studies to track the HRQL of individuals co-infected with HIV and SARS-CoV-2 over time would provide insights into the dynamic nature of HRQL in this population, including the impact of treatment regimens, viral load fluctuations, and potential long-term effects of co-infection.

## Data Availability

Data and materials used in the study for this manuscript are available from Dr Martin DURACINSKY (duracinsky.m@gmail.com).
